# Sutton on Cholera

**Published:** 1854-04

**Authors:** 


### SUTTON ON CHOLERA.

Dr. George Sutton, of Indiana, has reported to the Medical Society of that State on Cholera as it prevailed during the years 1859, 1852, inclusive, and has collected a body of facts which may be consulted with interest if that scourge should revisit us again, of which there seems to be but too much probability. It is a report of much labor, and appears to have been faithfully drawn up. Multiplied as are already the observations on that important subject, there is no danger that they will become too numerous, and all will find their place when the true theory of that strange disease comes to be expounded.

